# Factors associated with indication of prophylactic extraction of the lower third molar in orthodontic practice 

**DOI:** 10.4317/jced.61374

**Published:** 2024-03-01

**Authors:** Creysi Puyén-Goicochea, Mariano Ortiz-Pizarro, Daniel-José Blanco-Victorio, Victor Serna-Alarcón

**Affiliations:** 1 Private practice, Chiclayo, Peru; 2 Escuela de Medicina, Universidad César Vallejo, Trujillo, Peru; 3 Escuela de Medicina, Universidad Señor de Sipán, Chiclayo, Peru; 4 Escuela de Medicina, Facultad de Medicina, Universidad Privada Antenor Orrego, Trujillo, Peru

## Abstract

**Background:**

To date, there is no consensus on the factors that influence on indication for prophylactic extraction of the third molar, however it is a common indication in orthodontics. Aim: To determine the factors associated with indication of prophylactic extraction of the lower third molar in orthodontic practice.

**Material and Methods:**

This cross-sectional analytical study used an online survey to obtain responses from 100 professionals with clinical practice in orthodontics. The Survey Monkey software was used to enter a valid and reliable questionnaire of 11 questions to obtain demographic and clinical information of the professional, as well as some patient conditions that could be considered in a possible indication for prophylactic extraction. The questionnaire was sent through social networks and instant messaging applications. Chi Square test was used to evaluate associated factors and binomial logistic regression to identify risk or protective factors.

**Results:**

Factors significantly associated with indication of prophylactic extraction of the lower third molar were experience in orthodontics (*p*-value = 0.060; OR=0.325), characteristics of impaction (*p*-value = 0.012; OR=3.689), prevention of pericoronitis (p-value = 0.014; OR=3.769) and help stability of treatment results (*p*-value = 0.002; OR=6.074).

**Conclusions:**

The risk factors to indication for prophylactic extraction of the lower third molar were impaction of the third molar, prevention of pericoronitis and helping the stability of the results after treatment. Furthermore, experience in orthodontics was identified as a protective factor for this indication.

** Key words:**Orthodontics, risk factors, third molar, tooth extraction.

## Introduction

The third molar is a tooth characterized by certain variability in its presence or absence, in time of its conformation and calcification, its coronal and root morphology, as well as its eruption course and final position (1). Added to the above, in approximately 73% of people the lower third molar erupts in the third decade of life with an impaction rate that varies between 9.5% and 39% (2,3).

Among main problems that impacted third molars represent in relation to orthodontic treatment are possibility of direct or indirect interference in prevention or development of pathologies during treatment, in progress of treatment and posttreatment relapse (1,4). These reasons, added to the lack of solid evidence on the advisability of a prophylactic extraction, have caused the extraction of third molars to be a procedure indicated very frequently in patients who are going to receive orthodontic treatment (1,2).

The available literature recommends extraction under the criteria established in clinical practice guidelines for the benefit of treatment (5,6). All these recommendations agree on the need to extract a symptomatic or pathological third molar; however, there is no consensus on how to treat an asymptomatic patient (7). The prophylactic extraction of asymptomatic lower third molars is controversial, where it should be noted that “asymptomatic” does not rule out the possible existence of a disease or condition in progress (6). Professionals performing orthodontic treatment might recommend early prophylactic extraction of asymptomatic third molars to prevent the risk of future pathology and to minimize operative and postoperative risks. However, most third molars can erupt without associated symptoms (6,8). It should not be forgotten that numerous studies have addressed the different risks of third molar extraction such as: inferior alveolar nerve injury, mandibular angle fractures, postoperative infection, pain and functional limitation (9); in addition, to the lack of economic evidence to support the prophylactic extraction of mandibular third molars (10). Consequently, the recommendation to retain and monitor asymptomatic third molars can also be considered a valid option (8).

In this context, the professional who performs an orthodontic treatment makes the decision to indicate a prophylactic extraction or to preserve the third molar based on scientific information received, trends, preferences, experience, abilities and ethics. But factors external to the professional can also play a role, such as the characteristics of the patient, cultural, social and economic environment (5). Therefore, the objective of the present study was to identify whether factors such as years if professional experience, type of professional practice, mandibular growth characteristics, impaction characteristics of the third molar, caries prevention in the second molar, risk of pericoronitis, lower anterior crowding and stability of treatment results may be associated with the indication for prophylactic extraction of the lower third molar within orthodontic practice.

## Material and Methods

This analytical and cross-sectional study was approved by a postgraduate research committee through resolution No. 147-2021/EPGUSS-USS. Informed consent was obtained from all participants who agreed to answer a survey.

The participants were professionals registered in the College of Dentists of Peru and who frequently perform orthodontic treatment. The participants were general dentists or orthodontists; while that professionals who did not want to participate or who did not respond to the invitation to participate after two attempts were excluded.

The professionals were recruited through social networks, such as: Facebook®, Instagram®, Twitter®, LinkedIn® and WhatsApp®. The contacted professionals were asked if they frequently performed any orthodontic treatment in their clinical practice and were invited to participate in the survey regarding factors associated with the indication for prophylactic extraction of the lower third molar. The sample size was calculated using Freeman’s formula: 10 * (k + 1), where the value of K corresponds to the number of independent or predictive variables (11). The predictive variables were years of professional experience, type of professional practice, characteristics of mandibular growth, characteristics of impaction of the third molar, prevention of caries in the second molar, risk of pericoronitis, lower anterior crowding and stability of the results. Therefore, the sample size was 100 professionals, with a test power reached of 0.71, and snowball sampling was used to recruit study participants.

They were provided a link through the Survey Monkey® software to access the questionnaire. Each participant was asked for names of two other professionals who may be able to participate in the survey in order to improve recruitment. A first message or email was sent to potential participants, with a reminder after 7 days. The described strategy was used with purpose of contacting and enrolling to largest possible number of participants, since it was not possible to obtain a complete and updated list of personal data of professionals with clinical practice in orthodontics. The adherence rate of professionals in relation to the total number of questionnaires sent was 76.9%.

Data collection was carried out using a structured instrument of 11 closed questions with a dichotomous response alternative, was validated through participation of a team of five judges and reliability was determined using KR-20 test with a value of 0.903. The questionnaire containing three parts: a first for the presentation of the study and informed consent, the second regarding demographic data and the professional’s clinical practice, and a third related to the patient conditions patient and their possible influence on the indication for prophylactic extraction. Table 1 shows the questions and response alternatives used.

**Table 1:**
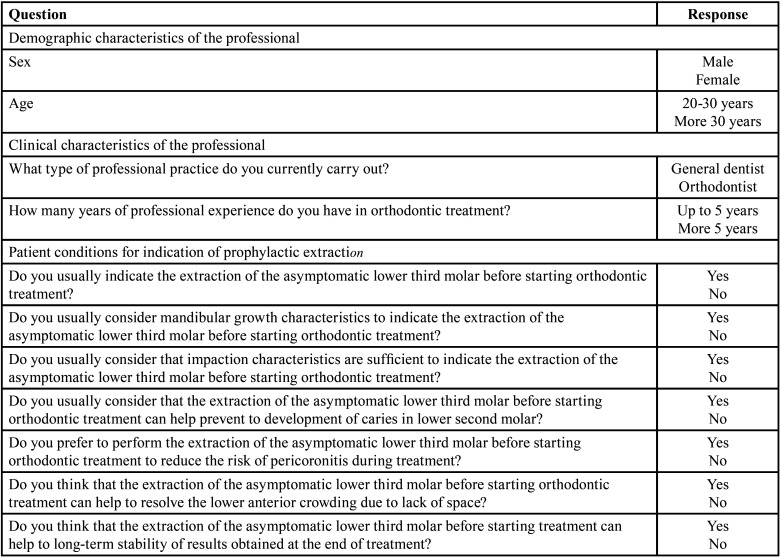
The questionnaire used to collect data in study.

-Statistical analysis

The statistical program STATA v17 (StataCorp, College Station, TX, USA) was used for data analysis. A bivariate analysis was performed to evaluate all possible factors associated with the indication for third molar extraction using Pearson’s Chi-square test. The Backward Stepwise method was used to determine the best logistic regression model. The final model allowed us to obtain the Odds Ratio (OR), the beta coefficients for each of the variables involved in the analysis. Establishing confidence intervals (CI) of 95% and a significance level of 5%.

## Results

A total of 100 professionals participated in the survey, 27 females and 73 males. Regarding age, 17 professionals ranged from 20 to 29 years, 33 professionals ranged from 30 to 40 years, and 50 professionals were over 40 years. The data presented in Table 2 show that demographic characteristics of the professionals did not influence the indication for prophylactic extraction of the lower third molar. With respect to the characteristics of clinical activity, the status of being a general dentist or orthodontists also did not influence the indication. However, professional experience was associated with a possible indication for prophylactic extraction.

**Table 2:**
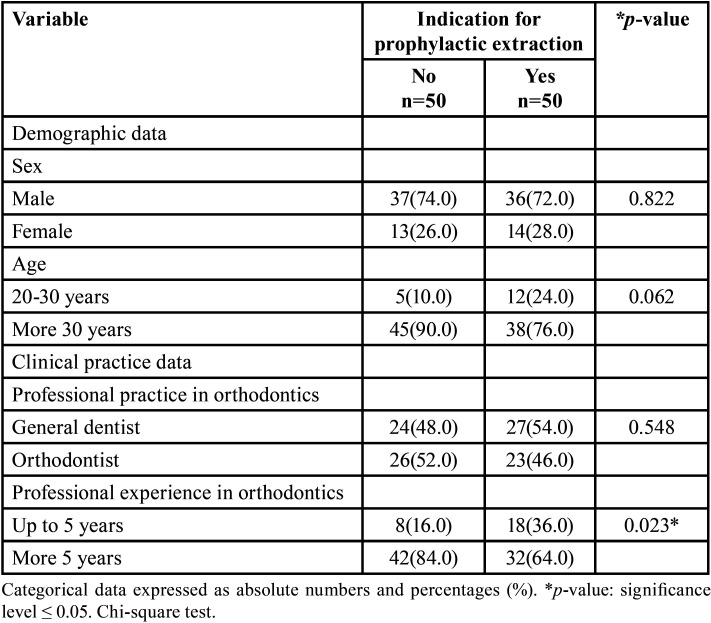
Demographic and clinical characteristics of professionals regarding the indication for prophylactic extraction of the lower third molar.

Table 3 shows the patient’s conditions and their influence on the indication for prophylactic extraction of the lower third molar. It was observed that the mandibular growth pattern and the type of impaction of the lower third molar were significantly associated with the indication for prophylactic extraction. The prevent development of pathologies related to the presence of the lower third molar was also evaluated. The indication for prophylactic extraction was not associated with prevention of caries in lower second molar. However, professionals did significantly associate their indication for prophylactic extraction of the lower third molar with possibility of preventing pericoronitis. The professionals significantly considered the possibility of prophylactic extraction of the third molar if the objective was to help resolve lower anterior crowding as a treatment outcome. Similarly, there was a significant association when considering prophylactic extraction with the purpose of improving the stability of long-term results.

**Table 3:**
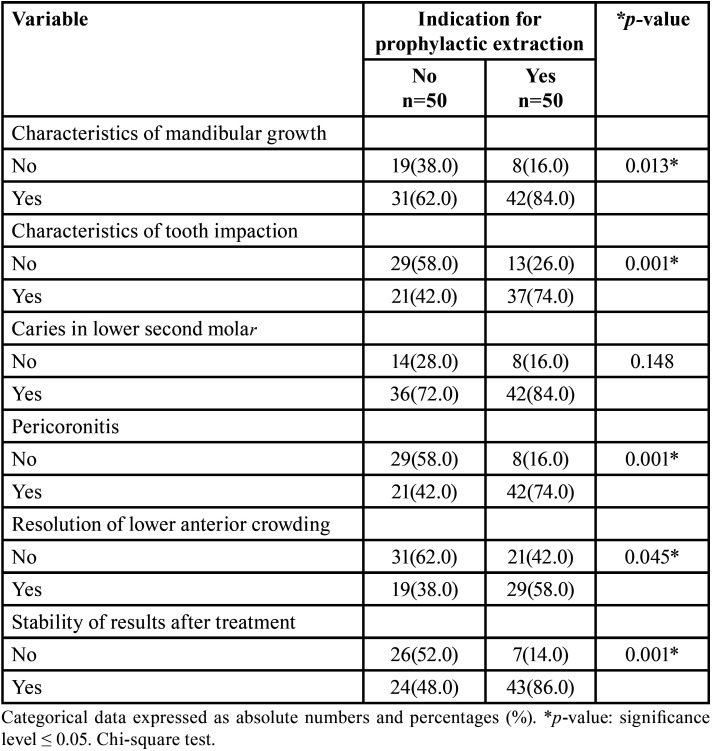
Patient conditions and their association with the indication for prophylactic extraction of the lower third molar.

Finally, Table 4 shows the resulting model in a conservative adjustment (pseudo R2 = 0.28), with statistical significance for factors or variables included (*p*=0.001). The data presented show sufficient evidence to affirm that risk factors associated with the indication for prophylactic extraction of the lower third molar are: characteristics of the impaction (OR = 3.69), prevention of pericoronitis (OR = 3.77) and contribution to stability of the results after treatment (OR = 3.07). Professional experience would mean a protective factor (OR = 0.33) for this indication by professionals who perform orthodontic treatment.

**Table 4:**
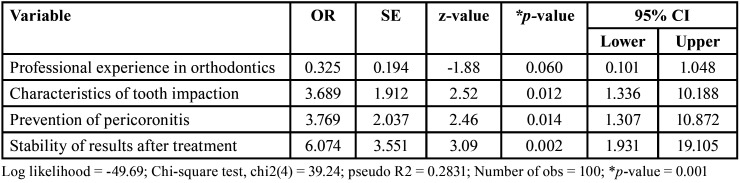
Factors associated with indication for prophylactic extraction of the lower third molar in orthodontic treatment – Logistic regression model.

## Discussion

In the present study, it was found that half of the professionals who participated in the survey would indicate the prophylactic extraction of an asymptomatic lower third molar before starting orthodontic treatment. This important percentage is close to that reported by Alfadil *et al*. in Saudi Arabia (12) and Alves-Pereira *et al*. in Spain (2). Although it should be noted that surveys used in these studies did not focus on the lower third molar which generally has a higher impaction rate neither in the context of orthodontic treatment, where the indication is more frequent (1,9). Previous studies have shown that the indication for extraction may be due to present symptoms or pathology, but also to prevent future problems generating controversy (8,13,14).

In analysis of the factors related to the dentist’s clinical practice, Sánchez-Jorge *et al*. found that the degree of training is related to the perception of a decision regarding the third molar (15), while Alnamlah *et al*. reports no association between the decision to perform a prophylactic extraction and being an orthodontist (16). This affirmation is in agreement with the results of the present study. Maybe because a specialist could be more informed about the still insufficient scientific evidence to justify this decision (2), and the general dentist could have a conservative attitude towards a prophylactic extraction based on professional experience (8,15). In this sense, antecedents found indicate that professional experience is an important factor in the indication of a procedure in relation to the lower third molar (2,15,16). These statements help to understand the significant association between the indication for prophylactic extraction and years of experience in orthodontic treatment. While the opposite occurred with the age and sex of the professionals, due to the lack of influence on a possible indication. A result that does not coincide with Alnamlah *et al*. that affirm a significant difference regarding both variables in a population of mostly male dentists and with a lower percentage of dentists over 40 years (16), which could be inconsistent with the role of professional experience (8,17).

In analysis of patient factors, professionals responded that mandibular growth significantly influences their decision to extract or not. A result that is supported in the literature because the lack of retromolar space or the availability of space for a correct location of the mandibular third molar is correlated with insufficient growth of the mandibular anatomy, which even depends on sex (5,18,19). For example, men’s jaws continue to grow during the eruption of third molars, while women’s jaws stop growing when third molars begin to erupt (19). Even the indications for germectomy of the mandibular third molar in class III patients have been described in cases where predictors of craniofacial growth reveal excessive anteroposterior mandibular growth, a severe dentoalveolar discrepancy or in cases where distalization of the first and second molars is necessary (20).

The association found between the characteristics of the impaction of the lower third molar and the decision to indicate a prophylactic extraction agrees with Alves-Pereira *et al*., De Sousa *et al*. and Cunha-Cruz *et al*. who found that impaction of the third molar is one of the factors more important that influences to a possible extraction of a lower third molar (2,5,8). The published literature indicates that a complex location of the third molar would affect the decision due to a positive correlation between the decision to extract a third molar and the probability of producing collateral damage (21), added to the fact that the professional’s ability to predict a risk or spontaneous eruption in cases of impaction has limitations (3,4,9).

One of the reasons evaluated in the survey was the indication for extraction for preventive reasons. Alves-Pereira *et al*. and Cunha-Cruz *et al*. mention that one of the reasons for professionals to indicate the extraction of a third molar is to prevent future problems such as cavities in the second molar (2,8), maybe as a consequence of the significant increase in bacterial plaque during orthodontic treatment (21). Even risk perception increases in third molars with greater inclination and with a horizontal position (2). However, in the present study no significant association was found in this regard, despite finding an impaction condition associated with the decision to extract. This non-influence is in accordance with a clinical and radiographic evaluation carried out by Kaye *et al*. who maintain that the extraction of a third molar is not justified because it does not represent a risk of losing the second molar (22); likewise, Alsaegh *et al*. report a relatively low frequency of second molar caries in cases of lower third molar impaction (21). Opposite results were observed for a significant association between the indication for prophylactic extraction and prevention of pericoronitis. Alves-Pereira *et al*., Cunha-Cruz *et al*. and Alfadil *et al*. report that pericoronitis is one of the main factors for a possible indication for extraction of asymptomatic third molars, and is even associated with greater patient adherence to this indication (2,8,12). However, the indication for extraction is still debated in cases of pericoronitis of limited duration related to a physiological movement of the third molar until it reaches a definitive position and that is independent even of orthodontic movements (5,8,23).

The results of the study show that the indication to extract a third molar was associated with the management of crowding before treatment. Alnamlah *et al*., Gavazzi *et al*. and Sharma *et al*. found that most orthodontists do not consider it useful to indicate third molar extraction to manage anterior crowding (16,17,24). Furthermore, there is longitudinal evidence to affirm that mandibular crowding is not influenced by the third molars (25). Although the literature is not conclusive in this regard, it has been stated that lower anterior crowding over the years may be due to other factors such as the tensile forces of the neighboring soft tissues, dimensions of the teeth, condition periodontal, evolutionary factors, sex and race of the patient (26). However, it should be considered that the responses in the present study were from orthodontic specialists and general practice dentists, compared to previous studies where only specialists participated. General practice dentists, due to limitations in training, are more likely to find a divergence between the perception of the need for extraction and the evidence that justifies the extraction, to finally rely on experience as an important factor in clinical decisions (8,15,27).

Professionals also associated the indication of prophylactic extraction with the objective of helping the stability of long-term treatment results. Alnamlah *et al*. shows that orthodontists do not consider third molar eruption as a cause of early relapse after treatment (16). In general, clinical studies have reinforced this position by demonstrating that relapse can occur regardless of the presence or absence of third molars (6,26). However, it has also been proposed that interproximal force may increase due to late eruption of the third molars, which would generate some movement in lower incisors, requiring constant evaluation after completing the retention phase (7,28).

Within the model obtained, it was found that the risk factors for prophylactic extraction are the impaction condition, pericoronitis and relapse after orthodontic treatment. While professional experience appears as a factor that protects against the extraction decision. So far, only Cunha-Cruz *et al*. present data from a regression analysis in general practice dentists from the PRECEDENT dental network of the Northwest, USA. Both studies agree in showing that the impaction conditions of the third molar are a risk factor for recommending its extraction (8). However, for American dentists, prevention of adverse conditions does not influence their recommendation for third molar extraction and neither does professional experience. The above is understandable because there are noTable differences in current practices around the world for cultural, sociodemographic and economic reasons (7,27); without forgetting the idiosyncrasy of patient, which can be very variable and can affect to acceptance of an initial indication of extraction provided by the professional prior to orthodontic treatment.

## Conclusions

The indication for a prophylactic extraction of the lower third molar was not associated with a condition of specialization in orthodontics, neither with the age and sex of professionals. However, this decision was associated with years of experience in orthodontic practice. Anatomical conditions such as mandibular growth and impaction characteristics influenced the indication for prophylactic extraction. A possible indication for extraction was associated with the intention to prevent pericoronitis, manage problems of lack of anteroinferior space and help the stability of long-term treatment results but not with the intention to prevent dental caries in the second molar. Evidence was found to affirm that the risk factors associated with the indication for prophylactic extraction of the lower third molar are: impaction characteristics, prevention of pericoronitis and help with the stability of treatment results; while years of experience in orthodontic treatments were observed as a protective factor for the indication for extraction.

## References

[1] Sifuentes-Cervantes JS, Carrillo-Morales F, Castro-Núñez J, Cunningham LL, Van Sickels JE (2021). Third molar surgery: Past, present, and the future. Oral Surg Oral Med Oral Pathol Oral Radiol.

[2] Alves-Pereira D, Pereira-Silva D, Figueiredo R, Gay-Escoda C, Valmaseda-Castellón E (2017). Clinician-related factors behind the decision to extract an asymptomatic lower third molar. A cross-sectional study based on Spanish and Portuguese dentists. Med Oral Patol Oral Cir Bucal.

[3] Libdy MR, Rabello NM, Marques LS, Normando D (2020). The ability of orthodontists and maxillofacial surgeons in predicting spontaneous eruption of mandibular third molar using panoramic serial radiographs. Dental Press J Orthod.

[4] Bastos Ado C, de Oliveira JB, Mello KF, Leão PB, Artese F, Normando D (2016). The ability of orthodontists and oral/maxillofacial surgeons to predict eruption of lower third molar. Prog Orthod.

[5] De Sousa AS, Neto JV, Normando D (2021). The prediction of impacted versus spontaneously erupted mandibular third molars. Prog Orthod.

[6] Peñarrocha-Diago M, Camps-Font O, Sánchez-Torres A, Figueiredo R, Sánchez-Garcés MA, Gay-Escoda C (2021). Indications of the extraction of symptomatic impacted third molars. A systematic review. J Clin Exp Dent.

[7] French Society of Stomatology, Maxillo-Facial Surgery and Oral Surgery SFSCMFCO (2020). French good practice guidelines regarding third molar removal: Indications, techniques, methods. J Stomatol Oral Maxillofac Surg.

[8] Cunha-Cruz J, Rothen M, Spiekerman C, Drangsholt M, McClellan L, Huang GJ; Northwest Practice-Based Research Collaborative in Evidence-Based Dentistry (2014). Recommendations for third molar removal: a practice-based cohort study. Am J Public Health.

[9] Petsos H, Fleige J, Korte J, Eickholz P, Hoffmann T, Borchard R (2021). Five-Years Periodontal Outcomes of Early Removal of Unerupted Third Molars Referred for Orthodontic Purposes. J Oral Maxillofac Surg.

[10] Hounsome J, Pilkington G, Mahon J, Boland A, Beale S, Kotas E (2020). Prophylactic removal of impacted mandibular third molars: a systematic review and economic evaluation. Health Technol Assess.

[11] Ortega Calvo M, Cayuela Domínguez A (2002). Regresión logística no condicionada y tamaño de muestra: una revisión bibliográfica [Unconditioned logistic regression and sample size: a bibliographic review]. Rev Esp Salud Publica.

[12] Alfadil L, Almajed E (2020). Prevalence of impacted third molars and the reason for extraction in Saudi Arabia. Saudi Dent J.

[13] Recchioni C, Junior ES, Ramacciato JC, Oliveira LB (2023). Oral maxillofacial surgeons and Orthodontists' perceptions about anterior inferior crowding and indications of mandibular third molar extraction. Med Oral Patol Oral Cir Bucal.

[14] Vranckx M, Fieuws S, Jacobs R, Politis C (2021). Prophylactic vs. symptomatic third molar removal: effects on patient postoperative morbidity. J Evid Based Dent Pract.

[15] Sánchez Jorge MI, Ocaña RA, Valle Rodríguez C, Peyró Fernández-Montes B, Rico-Romano C, Bazal-Bonelli S (2023). Mandibular third molar extraction: perceived surgical difficulty in relation to professional training. BMC Oral Health.

[16] Alnamlah S, Almazroa R, Alkhammash N, Alfotawi R, Premnath S (2021). Impacted Third Molars and Anterior Crowding-Beliefs and Evidence. J Res Med Dent Sci.

[17] Gavazzi M, De Angelis D, Blasi S, Pesce P, Lanteri V (2014). Third molars and dental crowding: different opinions of orthodontists and oral surgeons among Italian practitioners. Prog Orthod.

[18] Barone S, Antonelli A, Averta F, Diodati F, Muraca D, Bennardo F (2021). Does Mandibular Gonial Angle Influence the Eruption Pattern of the Lower Third Molar? A Three-Dimensional Study. J Clin Med.

[19] Bin Rubaia'an MA, Neyaz A, Talic F, Alkhamis A, Alghabban A, Assari A (2023). The Association Between Skeletal Facial Types and Third Molars Impaction in a Saudi Arabian Subpopulation: A CBCT Study. Clin Cosmet Investig Dent.

[20] Mazur M, Ndokaj A, Marasca B, Sfasciotti GL, Marasca R, Bossù M (2022). Clinical Indications to Germectomy in Pediatric Dentistry: A Systematic Review. Int J Environ Res Public Health.

[21] Alsaegh MA, Abushweme DA, Ahmed KO, Ahmed SO (2022). The pattern of mandibular third molar impaction and its relationship with the development of distal caries in adjacent second molars among Emiratis: a retrospective study. BMC Oral Health.

[22] Kaye E, Heaton B, Aljoghaiman EA, Singhal A, Sohn W, Garcia RI (2021). Third-Molar Status and Risk of Loss of Adjacent Second Molars. J Dent Res.

[23] Kang H, Lee NK, Kim J, Park JH, Kim Y, Kook YA (2021). Factors associated with the maxillary third molar position after total arch distalization using a modified C-palatal plate in adolescents. Orthod Craniofac Res.

[24] Sharma A, Reddy KK, Ramya Y, Betha SP, Tiwari RVC, Tiwari HD (2020). Effect of third molars in dental crowding as per orthodontist & oral surgeons perception: An original research. J Adv Med Dent Scie Res.

[25] Zigante M, Pavlic A, Morelato L, Vandevska-Radunovic V, Spalj S (2021). Presence and Maturation Dynamics of Mandibular Third Molars and Their Influence on Late Mandibular Incisor Crowding: A Longitudinal Study. Int J Environ Res Public Health.

[26] Lyros I, Vasoglou G, Lykogeorgos T, Tsolakis IA, Maroulakos MP, Fora E (2023). The Effect of Third Molars on the Mandibular Anterior Crowding Relapse-A Systematic Review. Dent J (Basel).

[27] Marya A (2022). Are orthodontic decisions consistent?. Evid Based Dent.

[28] Okazaki K (2010). Relationship between initial crowding and interproximal force during retention phase. J Oral Sci.

